# Effect of autonomic blocking agents on the respiratory-related oscillations of ventricular action potential duration in humans

**DOI:** 10.1152/ajpheart.00560.2015

**Published:** 2015-10-16

**Authors:** Stefan van Duijvenboden, Ben Hanson, Nick Child, Michele Orini, Christopher A. Rinaldi, Jaswinder S. Gill, Peter Taggart

**Affiliations:** ^1^Department of Mechanical Engineering, University College London, London, UK;; ^2^Department of Cardiology, Guy's and St. Thomas's Hospital, London, UK; and; ^3^Institute of Cardiovascular Science, University College London, London, UK

**Keywords:** respiration, cardiac electrophysiology, β-adrenergic blockade, parasympathetic blockade

## Abstract

Ventricular action potential duration (APD) is an important component of many physiological functions including arrhythmogenesis. APD oscillations have recently been reported in humans at the respiratory frequency. This study investigates the contribution of the autonomic nervous system to these oscillations. In 10 patients undergoing treatment for supraventricular arrhythmias, activation recovery intervals (ARI; a conventional surrogate for APD) were measured from multiple left and right ventricular (RV) endocardial sites, together with femoral artery pressure. Respiration was voluntarily regulated and heart rate clamped by RV pacing. Sympathetic and parasympathetic blockade was achieved using intravenous metoprolol and atropine, respectively. Metroprolol reduced the rate of pressure development (maximal change in pressure over time): 1,271 (± 646) vs. 930 (± 433) mmHg/s; *P* < 0.01. Systolic blood pressure (SBP) showed a trend to decrease after metoprolol, 133 (± 21) vs. 128 (± 25) mmHg; *P* = 0.06, and atropine infusion, 122 (± 26) mmHg; *P* < 0.05. ARI and SBP exhibited significant cyclical variations (*P* < 0.05) with respiration in all subjects with peak-to-peak amplitudes ranging between 0.7 and 17.0 mmHg and 1 and 16 ms, respectively. Infusion of metoprolol reduced the mean peak-to-peak amplitude [ARI, 6.2 (± 1.4) vs. 4.4 (± 1.0) ms, *P* = 0.008; SBP, 8.4 (± 1.6) vs. 6.2 (± 2.0) mmHg, *P* = 0.002]. The addition of atropine had no significant effect. ARI, SBP, and respiration showed significant coupling (*P* < 0.05) at the breathing frequency in all subjects. Directed coherence from respiration to ARI was high and reduced after metoprolol infusion [0.70 (± 0.17) vs. 0.50 (± 0.23); *P* < 0.05]. These results suggest a role of respiration in modulating the electrophysiology of ventricular myocardium in humans, which is partly, but not totally, mediated by β-adrenergic mechanisms.

## Abstract (precis)

Ventricular action potential repolarization is critical to electrical stability and arrhythmogenesis. Oscillations at the respiratory frequency were investigated in humans by combining endocardial electrophysiological recordings, controlled respiration with adrenergic blocking agents. Results are consistent with a partial role of the sympathetic nervous system combined with additional mechanisms, possibly involving mechano-electric feedback.

## NEW & NOTEWORTHY

Ventricular action potential repolarization is critical to electrical stability and arrhythmogenesis. Oscillations at the respiratory frequency were investigated in humans by combining endocardial electrophysiological recordings, controlled respiration with adrenergic blocking agents. Results are consistent with a partial role of the sympathetic nervous system combined with additional mechanisms, possibly involving mechano-electric feedback.

dynamic changes in action potential duration (APD) are a critical component of many fundamental electrophysiological properties. Alterations of the normal time course of APD are a key factor in arrhythmogenesis (25, 34, 51, 54). Elucidation of the multiple mechanisms that underlie modulation of ventricular repolarization is, therefore, an important challenge. Ventricular APD has recently been observed to exhibit oscillations related to respiration in humans with healthy ventricles (18, 19). The mechanism is as yet undetermined.

Several considerations suggest the possibility of a role of the autonomic nervous system in respiratory-related ventricular APD oscillations. Heart rate is well known to exhibit cyclical variation with the respiratory cycle, known as respiratory sinus arrhythmia, usually attributed to waxing and waning of autonomic input to the sinus node (1, 2, 4, 9, 31). Ventricular myocardium is now known to receive substantial vagal as well as sympathetic innervation (7), raising the possibility of cyclical autonomic influence in the ventricle. Both sympathetic and vagal stimulation may modulate ventricular APD (30, 32, 35, 43, 50, 55). Respiration is known to gate the timing of autonomic motor neurone firing such that inspiration is associated with an increase in sympathetic and decrease in parasympathetic nerve activity (8, 16, 28), again suggesting the possibility of respiratory-related autonomic modulation of ventricular APD. In the present study, therefore, we sought to examine the contribution of autonomic nervous system to respiratory-related ventricular APD oscillations in humans in vivo.

We have collected a unique database including activation recovery intervals (ARIs) measured from unipolar electrograms as a conventional surrogate for ventricular APD (5, 21, 33, 40, 53) from multiple right and left ventricular endocardial sites, femoral arterial blood pressure, and respiration (chest movement), in humans with healthy ventricles. Respiratory rate was controlled and heart rate clamped by ventricular pacing to avoid confounding effects due to the cycle length dependency of APD. Autonomic blocking agents were administered intravenously to selectively study the contribution of sympathetic and parasympathetic modulation of respiratory-related oscillations of APD. We hypothesized that autonomic blocking agents would reduce the amplitude of the oscillations if autonomic input is indeed involved. To further investigate the mechanisms underlying respiratory-related APD oscillations, multivariate frequency domain analysis was used to characterize the causal interactions between APD, respiration, and blood pressure. In addition, phase analysis was implemented to investigate the oscillatory behavior of APD in the left and right ventricle.

## METHODS

### 

#### Ethical approval.

The study was approved by the ethics committee of Guy's and Thomas′ Hospitals and conformed to the standards set by the Declaration of Helsinki (latest revision: 59th World Medical Association General Assembly). All patients gave written, informed consent.

#### Subjects.

Studies were performed in 10 patients (8 males, 2 females, aged 48–68, median 54) during the course of routine clinical radiofrequency ablation procedures for atrial fibrillation. Four patients had paroxysmal atrial fibrillation, and six patients established atrial fibrillation. All patients had normal ventricular function and were otherwise apparently healthy. No subject was known to have ventricular scar or disordered conduction due to bundle branch abnormality. The studies were conducted in the cardiac catheterization suite at St Thomas′ Hospital before the routine clinical procedure in the unsedated state as described previously (20, 45). Cardio-active medications were discontinued for 5 days before the study.

#### Measurements.

Synchronous measurements were made of the unipolar electrogram (UEG), femoral arterial blood pressure, and respiration (chest movement).

**Fig. 1.**
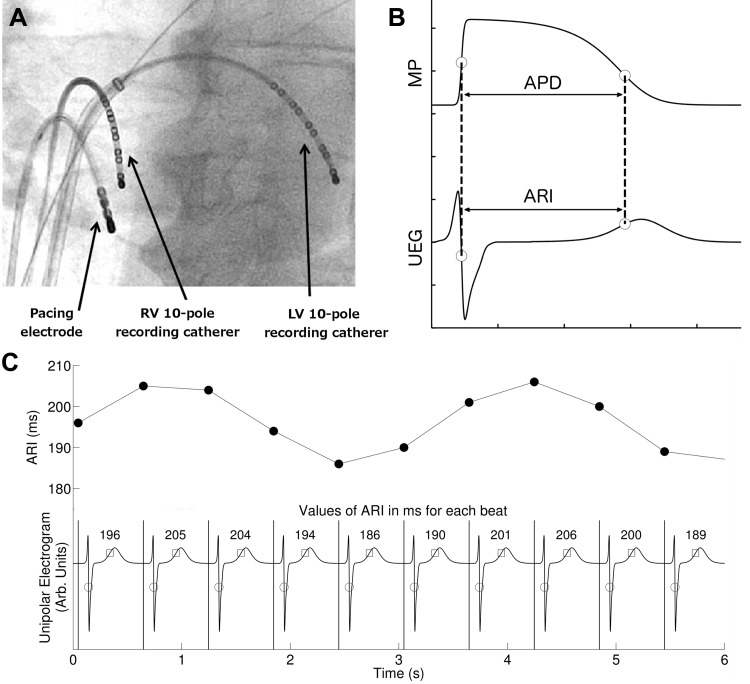
Electrophysiological measurements. *A*: fluoroscopic image showing the position of the two 10-pole recording catheters located in the left ventricle (LV) and right ventricle (RV). *B*: diagrammatic representation of the relationship between the unipolar electrogram (UEG) and the intracellular ventricular transmembrane potential (MP) during an action potential (AP) showing that the activation recovery interval (ARI) corresponds to the action potential duration (APD). *C*: example of ARI measurements of the local UEG: times of activation are marked with circles [minimal change in volume over time (dv/d*t*_min_) of the QRS] and repolarization with squares [maximal change in volume over time (dv/d*t*_max_) of the T wave]. The interval between the 2 points is the ARI, and values for each beat are shown in milliseconds. *Top*: corresponding ARIs plotted as function of time.

UEGs were measured using two decapolar electrode catheters (St Jude Medical, St. Paul, MN; 6F Livewire Steerable Catheter model 401915 with 2–5-2 mm spacing, 35 mm total span). One electrode catheter was introduced from the femoral vein into the left ventricle (via an atrial trans-septal approach) and positioned on the infero-posterior endocardial wall in a base-apex orientation. The other electrode catheter was introduced into the right ventricle and positioned on the anterior septal wall in a base-apex orientation. The electrode arrays were positioned over the mid- and lower third of the LV and RV endocardial wall. Both electrodes were referenced to a large skin surface electrode (100 × 150 mm) on the abdomen at the level of the naval such that distance to each individual electrode was considered to be approximately equal. The position of the recording and pacing electrodes are shown in Fig. 1*A*. Cine imaging fluoroscopy was used to verify secure positioning of the catheters throughout the cardiac and respiratory cycles. This has previously been established in detail for catheters in these positions (19).

Arterial blood pressure was measured from a femoral artery with a continuous-flush pressure transducer system (Tru-Wave PX600F; Edwards Lifesciences, Irvine, CA). The subject's breathing cycle was monitored using a custom-constructed tension sensor fixed to a freely expandable band placed around the chest/abdomen (adapted from a RESPeRATE device; InterCure, New York, NY). The optimum location for each subject was chosen as the site of maximum circumferential strain during normal breathing.

#### Controlled respiration.

To identify respiratory-related oscillations of ARI and SBP, subjects were instructed to breathe at four fixed rates (6, 9, 15, and 30 breaths/min) for 90 s each, in random order. This was achieved with the aid of a large video monitor with a 19-inch diagonal backlit screen mounted in easy line of site, on which was displayed a computer-generated animated visual display representing lung volume, which cycled at the required breathing rate (implemented in LabVIEW software; National Instruments, Austin, TX). Patients were instructed to breath at the same rate as the movement of the cursor on the screen. This was practiced before study commencement.

#### Protocol.

Subjects were paced from the right ventricular apex using a Biotronik (Berlin, Germany) stimulator (model UHS 3000) at 2× diastolic threshold and 2 ms pulse width, at a cycle length >20 beats/min faster than the intrinsic AF rate (median, 500 ms) to avoid breakthrough intrinsic beats and counteract the expected increase in intrinsic rate post atropine. A 2-min period of adaptation to the paced cycle length was applied before starting the controlled breathing protocol. First, a control period was established with the breathing protocol performed in absence of any autonomic blocking agents. The subject then received metoprolol at a dose sufficient to reduce the intrinsic heart rate by >10 beats/min (iv; dose range, 2–10 mg), and after ∼10 min for equilibration the breathing protocol was repeated. Finally, the subjected was given atropine at a dose sufficient to increase the intrinsic heart rate >10 beats/min faster than the starting heart rate (iv; dose range, 600–1200 μg), and the breathing protocol was once again repeated. During the experiment, electrograms, blood pressure, and respiration were recorded synchronously.

#### Data analysis.

Electrogram, blood pressure, and respiration recordings were sampled at 1,200 Hz (Ensite 3000; Endocardial Solutions) and analyzed offline. Ventricular APDs at each recording site were estimated from the UEG by measuring activation-recovery intervals (ARIs) using the Wyatt method (53). This method has been validated in theoretical, computational, and experimental studies (5, 21, 33, 40, 52). According to this method, activation is measured at the moment of minimum dV/d*t* of the QRS complex of the UEG (5, 21, 33, 40, 53) and repolarization at the moment of maximum dV/d*t* of the T-wave. The relationship between APD and ARI is illustrated in Fig. 1*B*. In this work, ARI was measured semi-automatically using a custom algorithm written in MATLAB (Matlab Mathworks). Heuristic-based screening was used to identify and discount any cases where the T-wave was indistinct or corrupt.

Blood pressure recordings were analyzed for systolic blood pressure (SBP) and the maximum rate of systolic pressure increase (dP/d*t*_max_) as a measure of myocardial contractility. To establish evenly sampled series, any beats for which ARI or SBP measurement could not be determined were replaced by linear interpolation between the surrounding beats. If these surrogate beats constituted more than 10% of any series, the series was rejected.

#### Amplitude estimation of respiratory-related ARI and SBP oscillations.

The peak-to-peak amplitude of respiratory-related ARI and SBP oscillations, hereinafter referred to as oscillations, was estimated in the frequency domain using the Thomson's multitaper method with three Slepian tapers (48). This method was used because of its robustness against noise. The power inside a breathing frequency band, defined as the breathing frequency ± 10%, was measured and then converted to amplitude, which is half the peak-to-peak amplitude. The ARI and SBP peak-to-peak amplitude was measured in milliseconds and millimeters of mercury, respectively. The breathing frequency was defined as the peak frequency in the power spectrum of the respiratory signal.

Surrogate data analysis was used to establish a threshold to test whether ARI oscillations were significant. Surrogate data series were created by random permutations of the signal such that the amplitude distribution remained intact, but the original (oscillatory) behavior was destroyed (14). For each signal, 1,000 surrogate series were generated and their corresponding spectra were computed. The threshold function for significance was then determined for each frequency bin as the upper 95th percentile of the surrogate distribution. ARI oscillations were considered significant if the power at the breathing frequency exceeded this threshold (*P* < 0.05). Recording sites that showed significant oscillations were selected for further analysis to investigate the effect of autonomic drugs on the amplitude of the oscillations.

#### Assessment of coupling and causality.

Interactions between ARI, SBP, and respiration (RESP) were characterized in the frequency domain. Coupling was studied by means of coherence, which quantifies the coupling strength between two signals as a function of frequency. The coherence attains a value between 0, indicating absence of coupling, and 1, indicating full coupling. Directed coherence was used to infer causality. Intuitively, the directed coherence represents the fraction of the power spectrum of a signal due to another signal through direct or indirect pathways. This measure of causality is grounded on the notion of Granger causality, stating that a process is causal to another if the prediction of the second is improved by incorporating the knowledge of the first (13). In this work, both coherence and directed coherence are formulated in the framework of an extended linear multivariate autoregressive (MVAR) model proposed (11–13). This model takes into account both instantaneous and lagged effects. The MVAR model is defined in Appendix A. Definitions for coherence and directed coherence are provided in Appendix B. The coefficients of the (multivariate) model were estimated using the least-squares approach with a fixed model order of 10. The resulting residuals of the trivariate model were tested for white noise and independence.

#### Phase relationship between ARI oscillations in the left and right ventricle.

The phase relationship between left and right ventricular ARI oscillations was studied by computing the mean phase lags for the left and right ventricle recording sites. The lag was measured using the Thomson's multitaper cross-power spectrum computed to determine the phase at the breathing frequency using three tapers (23).

Recordings sites were included only if the signal exhibited significant amplitude and coherence at the breathing frequency. The relationship was then investigated by subtracting the mean phase from the left and right ventricle.

#### Statistical analysis.

For all subjects, mean values obtained for dP/d*t*_max_, SBP, amplitude of ARI, and SBP oscillations and the (directed) coherence measures were averaged across the four respiratory rates. The effect of β-blocker and atropine infusion was then investigated by comparing control and β-adrenergic blocking (BB) values and BB and BB + atropine (AT) values using the two-tailed paired Wilcoxon signed-rank test for statistical significance. Results were considered significant at *P* < 0.05.

## RESULTS

### 

#### The effect of autonomic blocking agents on blood pressure.

**Fig. 2.**
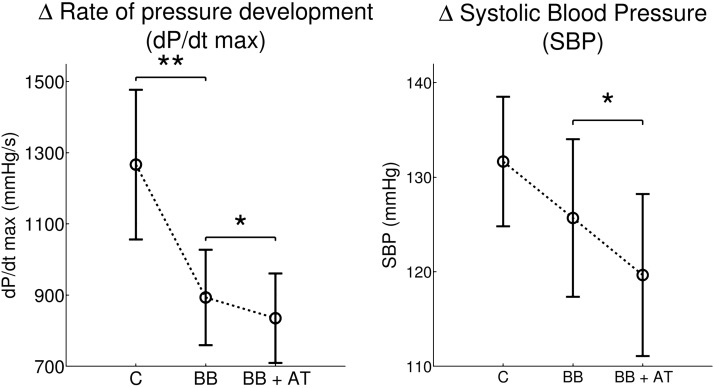
*Left*: maximum rate of systolic pressure increase (dP/d*t*_*max*_) was significantly reduced after administration of the β-adrenergic blocking (BB) agent metoprolol. Subsequent addition of atropine (AT) was associated with a further small but significant reduction. *Right*: systolic blood pressure (SBP) showed no significant change following metoprolol but a significant reduction following the addition of atropine. Error bars indicate SE. C, control. *P < 0.05; **P < 0.01.

Figure 2 shows the effect of autonomic inhibitors on mean blood pressure indexes. After administration of β-adrenergic blocking agents (metoprolol), the mean dP/d*t*_max_ was significantly decreased [1,271 (± 646) vs. 930 (± 433) mmHg/s; *P* < 0.01]. The mean SBP showed a tendency to decrease [baseline 133 (± 21) vs. metoprolol 128 (± 25) mmHg; *P* = 0.06]. Addition of atropine was associated with a further significant decrease of the mean dP/d*t*_max_ [930 (± 433) vs. 887 (± 436) mmHg/s; *P* < 0.05]. The mean SBP showed also a significant decrease following the addition of atropine: 128 (± 25) vs. 122 (± 26) mmHg; *P* < 0.05.

Significant ARI and SBP oscillations were observed in all subjects at all breathing frequencies in 61% and 92% of the available recordings, respectively.

**Fig. 3.**
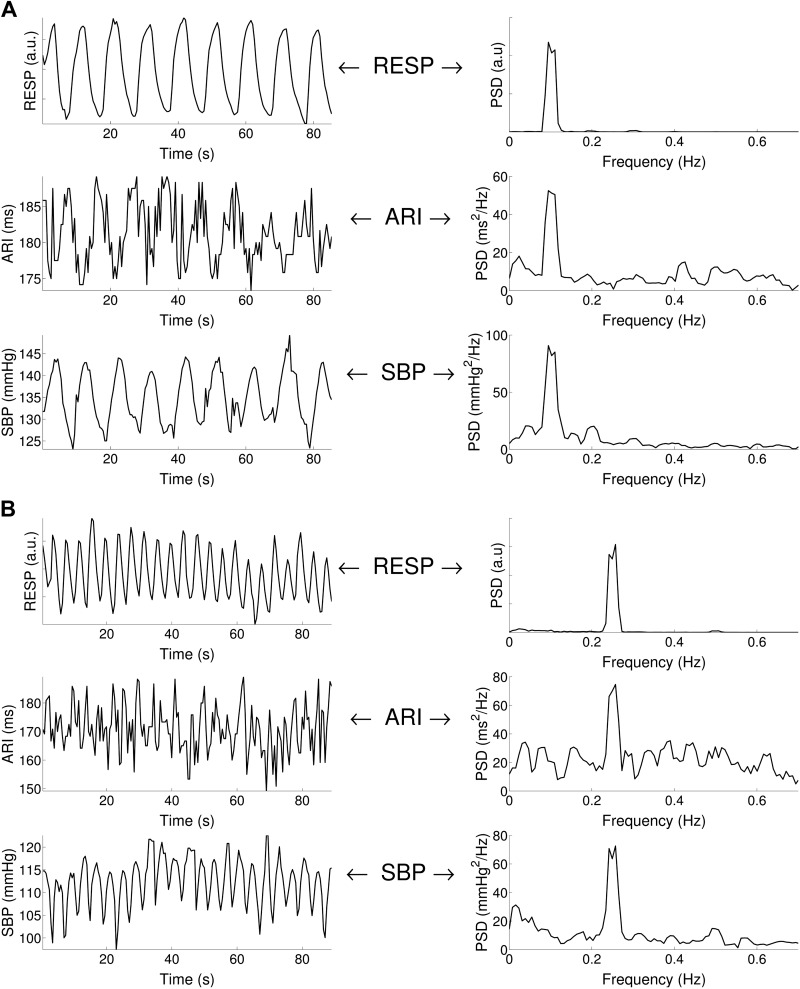
Respiratory-related ARI and SBP oscillations. *A*: example of ARI, SBP, and respiration (RESP) recordings for 0.1 Hz (6 breaths/min) breathing. Cyclical variation was observed in both ARI and SBP at the breathing frequency. PSD, power spectral density. *B*: example of ARI, SBP, and RESP recordings for 0.25 Hz (15 breaths/min) breathing. Cyclical variation was observed in both ARI and SBP at the breathing frequency.

Two examples of ARI and SBP time series during paced breathing at 6 and 15 breaths/min are shown in Fig. 3. In both cases, ARI and SBP exhibit oscillatory behavior at the breathing frequency.

**Fig. 4.**
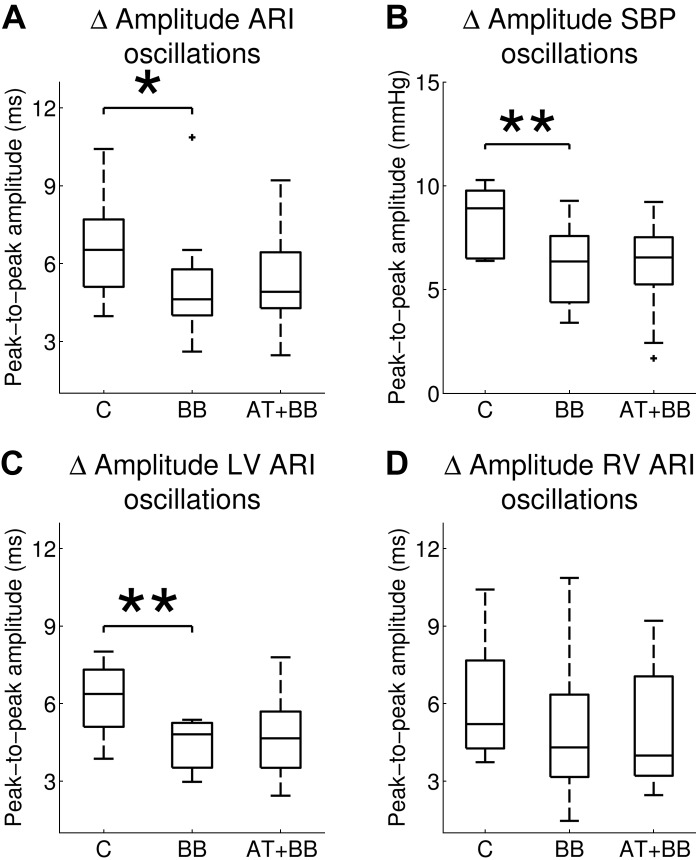
Mean values of peak-to-peak amplitude of respiratory-related oscillations of ARI and SBP during control (C), following administration of β-adrenergic blocking (BB) agents and after subsequent addition of atropine (AT + BB). After administration of β-blockers, the amplitude of LV ARI and SBP oscillations were significantly reduced, and a trend to reduction was seen for RV ARI oscillations. *P < 0.05; **P < 0.01.

The peak-to-peak amplitude of ARI oscillations was ranging from 1.0 to 16.0 ms (left ventricle, 1.0–12.9 ms; right ventricle, 1.0–16.0 ms). The effect of autonomic blocking agents on the mean peak-to-peak amplitude of respiratory-related ARI and SBP oscillations is shown in Fig. 4. After administration of β-adrenergic blockers, the mean ARI peak-to-peak amplitude decreased [6.6 (± 1.9) vs. 5.1 (± 2.4) ms; *P* = 0.04; Fig. 4*A*]. The main effect was in the left ventricle, which showed a significant reduction in peak-to-peak amplitude [6.2 (± 1.4) vs. 4.4 (± 1.0) ms; *P* = 0.008; Fig. 4*C*]; in the right ventricle the differences were not statistically significant [6.0 (± 2.2) vs. 4.9 (± 2.7) ms; *P* = 0.2; Fig. 4*D*]. Addition of atropine did not result in any further changes of the ARI peak-to-peak amplitude in both left and right ventricle.

The peak-to-peak amplitude of SBP oscillations was ranging from 0.7 to 17.0 mmHg. The mean peak-to-peak amplitude of SBP oscillations showed a significant reduction following the administration of β-adrenergic blockers [8.4 (± 1.6) vs. 6.2 (± 2.0) mmHg; *P* = 0.002; Fig. 4*B*]. Subsequent addition of atropine was not associated with any significant change [6.2 (± 2.0) vs. 6.0 (± 2.4) mmHg; *P* = 0.8].

In the LV the absolute ARI shows a tendency to increase at the sites where ARIs were measured after administration of the β-blocker: control, 189 ms (± 25 ms), β-blocker: 191 ms (± 23 ms); this would be consistent with a reduced sympathetic action on APD. Subsequent atropine administration shortened ARI to 189 (± 20 ms), consistent with reduced parasympathetic restraint on residual sympathetic tone resulting in APD shortening.

#### Assessment of the interactions between ARI, SBP, and respiration.

**Fig. 5.**
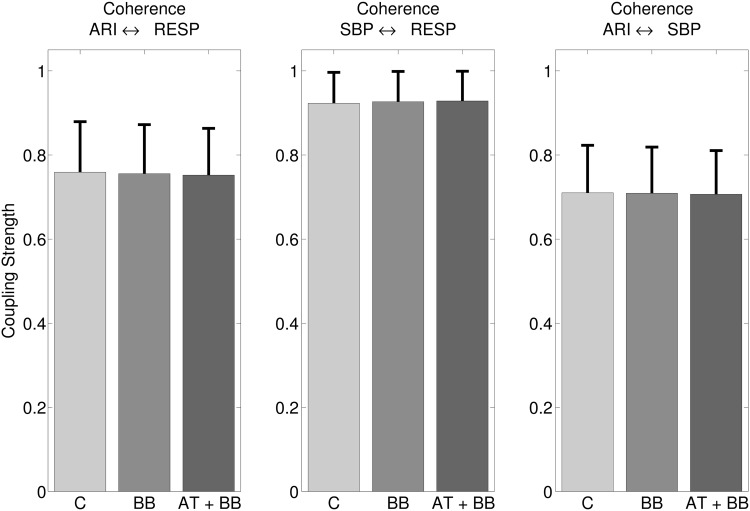
Mean coherence measures the coupling strength between ARI and respiration (*left*), between SBP and RESP (*middle*), and between ARI and SBP (*right*). Significant coherence was detected in all subjects and was unaffected after administration of autonomic blocking agents.

Significant coherence (*P* < 0.05) was detected between ARI, SBP, and RESP, significant in all subjects. The coherence was significant in 47% of the ARI signals. Figure 5 shows the mean coherences between ARI, SBP, and RESP. For example, Fig. 5, *left*, shows that the mean coherence (coupling strength) between ARI and RESP was strong, being 0.76 on a scale of 0 to 1. The mean coherence was not affected by the administration of autonomic inhibitors: ARI ⇔ RESP: 0.76 (± 0.12) vs. 0.76 (± 0.12) vs. 0.75 (± 0.11); ARI ⇔ SBP: 0.71 (± 0.11) vs. 0.71 (± 0.11) vs. 0.71 (± 0.11); SBP ⇔ RESP: 0.92 (± 0.07) vs. 0.93 (± 0.07) vs. 0.93 (± 0.07) for control, β-blocker, and subsequent addition of atropine.

**Fig. 6.**
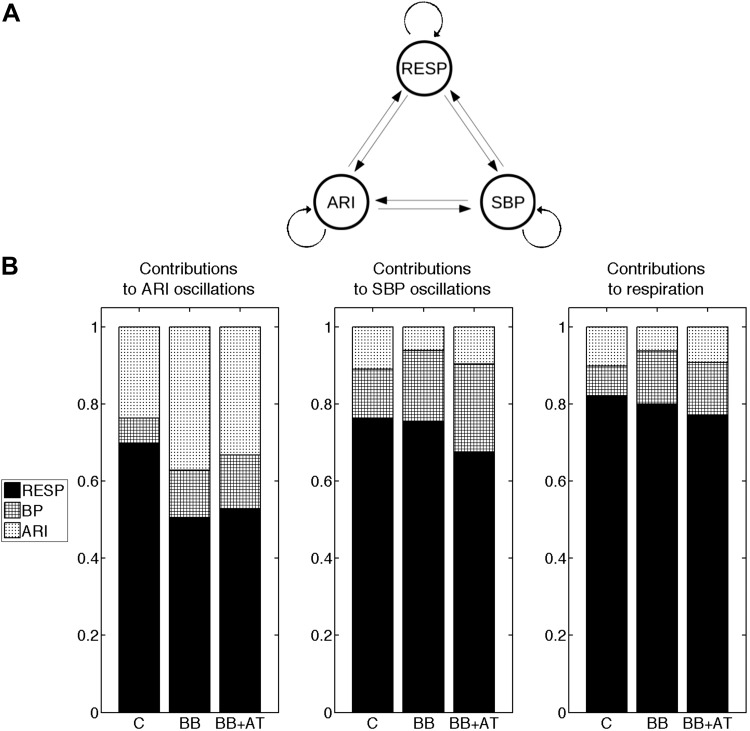
Assessment of the causal interactions between respiration (RESP), ARI, and SBP. *A*: possible (theoretical) interactive pathways between all processes. *B*: for all processes the mean relative power contributions related to all processes (the directed coherence) at the breathing frequency. Black quantifies the contribution of respiration; densely hatched represents SBP and lightly hatched ARI. The directed coherence was measured during control (C), following administration of β-adrenergic blocking (BB), and subsequent addition of atropine (BB + AT). For example, during control, the directed coherence from respiration to ARI (*left*) is 0.70, indicating that respiration explains 70% of ARI signal power at the breathing frequency. Administration of β-blocker agents significantly reduced the contribution of respiration to respiratory-related oscillations of activation recovery intervals (*P* < 0.05).

The results of the directed coherence analysis are presented in Fig. 6. Figure 6*A* shows a graphical representation of the (theoretical) possible interactive pathways between all processes. The directed coherence describes the coherence according to the direction of information transmission by measuring the relative power contributions for all processes (ARI, SBP, and respiration). Figure 6*B* shows the mean directed coherence at the breathing frequency during control, β-blocker, and subsequent addition of atropine. Regarding the observed ARI oscillation, during control, the directed coherence from respiration to ARI (RESP ⇒ ARI) was 0.70 (± 0.17), which means that, at the breathing frequency, on average, 70% of the ARI signal power could be explained by the respiratory signal.

In contrast, the directed coherence from SBP to ARI (SBP ⇒ ARI) was found to be marginal: SBP ⇒ ARI: 0.07 (± 0.06), indicating that the causal interaction from SBP to ARI was weak.

High directed coherence was also found from respiration to SBP (RESP ⇒ SBP): 0.70 (± 0.17), whereas, as expected, the directed coherence from ARI to SBP (ARI ⇒ SBP) was low: 0.11 (± 0.05).

After administration of β-adrenergic blockers, the directed coherence from respiration to ARI was significantly reduced: 0.70 (± 0.17) vs. 0.50 (± 0.23), *P* < 0.05. The directed coherence from SBP to ARI showed a tendency to increase, but remained very small: SBP ⇒ ARI: 0.07 (± 0.06) vs. 0.12 (± 0.07), *P* = 0.06.

Regarding SBP oscillations, RESP ⇒ SBP was not affected by β-blocker: 0.76 (± 0.17) vs. 0.75 (± 0.11), *P* = 0.9. When compared with RESP ⇒ SBP, ARI ⇒ SBP was very small during both control and β-blocker: 0.11 (± 0.05) and 0.06 (± 0.06), respectively (*P* < 0.05). Subsequent addition of atropine did not result in any further changes of the directed coherences (RESP ⇒ ARI, SBP ⇒ ARI, RESP ⇒ SBP, and ARI ⇒ SBP).

#### Investigation of the phase of left and right ventricular ARI oscillations.

**Fig. 7.**
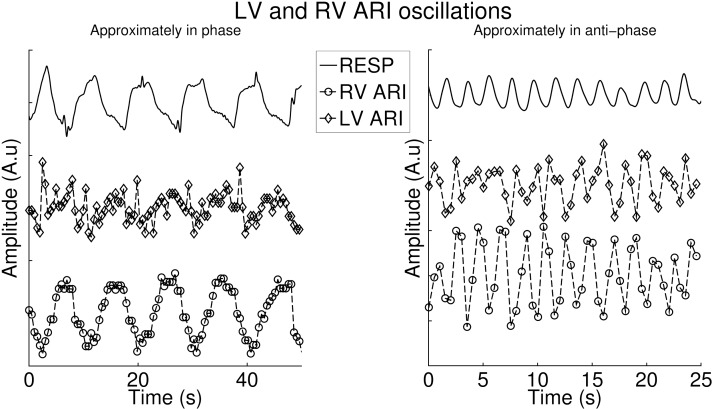
Two examples of oscillations in the RV and LV. *Right*: example in which both oscillations are approximately in phase. *Left*: example in which the oscillations are approximately in anti-phase. Note the different time scales for clarity.

The phase lag between respiration and ARI was investigated for left and right ventricular recordings. During control, on average, ARI oscillations in the LV lagged behind RV oscillations: the mean relative phase difference was statically different from 0: 37 (± 46) dgr, *P* < 0.05. The phase difference did not change after administration of β-blocker: 37 (± 46) vs. 17 (± 45) dgr, *P* = 0.6. However, the phase difference was not statically significant from 0 anymore (*P* = 0.2). Addition of atropine was not associated with additional changes: 17 (± 45) vs. −4 (± 78), *P* = 0.7. Various phase differences between RV and LV were observed between subjects. Figure 7 shows an example of LV and RV ARI signals that are approximately in phase (Fig. 7, *left*) and in anti-phase (Fig. 7, *right*).

## DISCUSSION

The duration of the ventricular action potential plays a crucial role in maintaining electrical stability and in arrhythmogenesis. Oscillations of APD in ventricular myocardium have recently been shown to occur at the frequency of respiration in human subjects (18, 19). To investigate the underlying mechanism, we have used infusion of autonomic blocking agents together with causal coherence analysis in subjects with healthy ventricles while recording a measure of ventricular endocardial APD using catheter electrodes, SBP, and respiration. The main findings were as follows: *1*) we confirmed the presence of oscillations in APD and SBP at each of four controlled respiratory frequencies: 6, 9, 15, and 30 breaths/min; *2*) the β-adrenergic blocking agent metoprolol resulted in a decrease in the amplitude of APD oscillation; *3*) IV administration of atropine following metoprolol was without effect on LV or RV ARI oscillation amplitude; *4*) coherence analysis showed a significant linear coupling between respiration, APD, and SBP at the breathing frequency; *5*) analysis of the directed coherence showed a high directed coherence from respiration to APD and from respiration to SBP. β-Adrenergic blockade reduced the contribution of respiration to APD oscillations.

### 

#### Methodological considerations.

The unipolar signal recorded using the multipolar catheters as used here (St. Jude Medical) has been extensively validated as a true representation of the local endocardial activity, and the derived ARI measure as representative of the local APD (5, 40, 53), and has been further validated in the context of the protocol used in the present study (52). Respiratory frequency was well controlled by the subjects, but we did not measure tidal volume, arterial P_CO2_, and pH, which may affect APD. However, we measured blood gasses and discussed this issue in a previous communication and concluded that any possible influence would be small (19). In these studies autonomic blockade was not complete. The dosage of metoprolol and atropine was titrated on an individual patient basis to achieve a target change in heart rate. This is a regime used in the clinical scenario where complete blockade is avoided as the border between complete blockade and overdose is narrow and unpredictable. As a result, we cannot exclude the possibility that the level of blockade may have affected our results. It is well known that there is regional variation in parasympathetic innervation, which is greatest at the base and decreases toward the apex, as well as epicardial to endocardial differences. The electrode arrays were located over the mid- and lower third of each ventricle where stable recordings are best obtained. It is possible, therefore, that a response to atropine occurred in the more basal regions, which we did not detect.

#### APD oscillations-mechanisms.

We are at present unaware of any reported observations on the effect of autonomic blocking agents on the respiratory-related oscillations of ventricular APD. Respiration physically alters membrane potentials of sympathetic and parasympathetic preganglionic motoneurones and thereby continuously modulates sympathetic neuronal and vagal motorneurone activity over a wide range of frequencies (8), both of which are known to modulate APD (30, 32, 35, 50, 55). Our results show a reduction in respiratory-related ARI oscillation following administration of the β-blocking agent metoprolol with further reduction following atropine, suggesting a role for autonomic nerve activity. One possibility is a baroreflex-mediated effect on the autonomic modulation of individual myocytes and hence APD in response to cyclic variation in the hemodynamics during the respiratory cycle. Although the exact relation between cardiopulmonary hemodynamics and right and left ventricular pressure-volume relations has been the subject of debate, a general consensus is that during inspiration the fall in intrathoracic pressure results in increased filling of the right heart with increased right ventricular stroke volume. The increased pleural pressure increases left ventricular afterload and together with the increased right ventricular volume result in reduced stroke volume during inspiration (17, 24, 26, 41, 49). In RR interval studies, it has been shown that the influence of SBP variability on RR variability was high during rest (36–39). The methodology used in this work did not reveal a strong causal interaction from SBP to ARI respiratory oscillations (Fig. 6). This should be interpreted within the limit of a linear model and does not exclude a more complex involvement of blood pressure in the generation ARI oscillation. For example, a change in intraventricular pressure may induce regional changes in cardiac strain that could in turn cause ARI oscillations via the mechano-electric feedback pathway (44, 46). Therefore, blood pressure changes, which only partially reveals heterogeneous cardiac strain, may be involved in the generation of ARI oscillations without necessarily exhibiting a linear interaction with them.

Another possible mechanism is central gating of autonomic neural traffic by central respiratory networks (15), arising as a result of brainstem interactions (6) or entrained by cortical activity during controlled breathing (10). We could not exclude the possibility that the cardioselective β-adrenergic blocking agent metoprolol we used, being moderately lipophilic and therefore capable of passing the blood brain barrier, could have exerted a central effect.

Parasympathetic activity decreases during inspiration and, therefore, could potentially influence APD by the ACh-activated K^+^ current IK, ACh, now recognized as being widely represented in ventricular myocardium (7). In the present study the administration of atropine after β-blockade induced no further effect on the amplitude of the ARI oscillations, suggesting no significant involvement of this current.

The β-adrenergic signaling cascade involves phosphorylation of a number of target proteins, and it is at present unclear as to whether the time constants of the phosphorylation/dephosphorylation process would be capable of following the breathing frequencies used in the present study. An alternative possibility is that sympathetic activity may act indirectly by interacting by modulating mechano-electric coupling (MEC).

MEC whereby changes in myocardial fiber stretch/strain alter the electrophysiology (29, 44, 46) has been shown to be substantially enhanced by β-adrenergic blocking agents (22), and therefore the reduction in the oscillation of APD that we observed after administration of metoprolol would be consistent with MEC as an underlying mechanism. The effects of MEC on APD are complex depending on the nature and timing of the mechanical perturbation (3, 27, 42, 47). In general increased stretch tends to shorten APD. Increased fiber length increases the affinity of Troponic C for calcium, which slows calcium release from TnC resulting in a reduced calcium transient, decreased NaCa exchange current, and APD shortening. Increased fiber shortening results in a decreased affinity of TnC for calcium, faster calcium release, an increased calcium transient, increased inward NaCaX, and APD prolongation. In addition stretch-activated channels either shorten or lengthen APD dependent on the timing of the stretch relative to the reversal potential (∼ −30 mv), such that early stretch shortens and late stretch lengthens APD. Thus the overall effect is likely to be a complex function of whichever mechanism is dominant. As described above, during inspiration LV stroke volume decreases, which would be expected to reduce stretch and fiber excursion, whereas opposite effects would be expected in the RV. The marked variability that we observed in phase of ARI oscillations that we observed between endocardial left ventricular free wall and right ventricular septum would be consistent with such a mechanism. Discordant ARI oscillations in the LV and RV increased the regional ARI differences between LV and RV. We observed that the dispersion of ARI (APD) between left and right ventricle could increase up to ∼40 ms when ARI oscillations reached their peak in the RV and its trough in the LV at the same time. Because ARI is a component of total repolarization and regional differences in repolarization are important in arrhythmias based on reentry, the cyclical variation in dispersion of ARI due to respiration may contribute to the conditions for reentry to occur.

### Conclusion

The dynamics of ventricular repolarization play a critical role in maintaining electrical stability. We have investigated the role of the autonomic nervous system in generating oscillations in ventricular APD at the respiratory frequency in humans with healthy ventricles. Ventricular APD and SBP exhibited oscillations at each of four controlled respiratory frequencies. The β-adrenergic blocking agent metoprolol partly but not completely reduced the APD oscillation. The addition of a parasympathetic blocking agent (atropine) was without any additional effect. Directed coherence as a measure of causality indicated respiration rather than (femoral artery) SBP as a major cause of APD oscillation. β-Adrenergic blockade reduced the contribution of respiration to APD oscillations. The results are consistent with a role of the sympathetic nervous system combined with an additional mechanism.

## GRANTS

S. van Duijvenboden is supported by the UCL Impact bursary scheme. M. Orini is supported by the IEF-2013 Marie Curie Fellowship. B. Hanson is in receipt of a grant from the UK Medical Research Council MRC Project Grant (Ref G0901819).

## DISCLOSURES

No conflicts of interest, financial or otherwise, are declared by the author(s).

## AUTHOR CONTRIBUTIONS

Author contributions: S.v.D. analyzed data; S.v.D., B.H., M.O., and P.T. interpreted results of experiments; S.v.D. prepared figures; S.v.D., M.O., and P.T. drafted manuscript; S.v.D., B.H., N.C., M.O., C.A.R., J.G., and P.T. edited and revised manuscript; S.v.D., N.C., M.O., and P.T. approved final version of manuscript; B.H., M.O., J.G., and P.T. conception and design of research; B.H., N.C., and J.G. performed experiments.

## Appendix A: MULTIVARIATE AUTOREGRESSIVE MODEL

In this study, ARI, SBP, and RESP are a joint multivariate zero-mean process: Y(n) = [y_ARI_(n), y_SBP_(n), y_RESP_(n)] ^T^. Here, ARI is numbered as process 1, SBP is process 2, and RESP is process 3. The set was described as a multivariate autoregressive (MVAR) process defined as:

$$Y\left(n\right)=\sum_{k=1}^{p}A\left(k\right)Y\left(n-k\right)+U\left(n\right)$$

where p is the model order, A(k), k = 1, …, p, are the 3×3 matrices containing the coefficients a_ij_(k) that describe the linear interaction at lag k from y_j_(n-k) to y_i_(n) (i,j = 1,2,3), and U(n) = [u_1_(n), u_2_(n), u_3_(n)]^T^ is a vector of zero-mean white noise processes with diagonal covariance matrix ∑_U_. This strictly causal model representation (k>0) cannot describe zero-delay correlations among the observed series Y, which are thus explained by correlations among the input noises U. To overcome this problem, series Y can be described including instantaneous effects [y_i_(n) to y_j_(n)] into the interactions allowed by the model as proposed by Faes and Nollo (12). This is achieved by extending the MVAR process:

$$Y\left(n\right)=\sum_{k=0}^{p}B\left(k\right)Y\left(n-k\right)+W\left(n\right)$$

Like U(n), W(n) = [w_1_(n), w_2_(n), w_3_(n)]^T^ is a vector of uncorrelated processes with diagonal covariance matrix ∑_W_. Instantaneous effects are modelled in the form of the coefficients b_ij_(0) of the matrix B(0). B(0) is a lower triangular matrix with null diagonal and can only be solved by imposing a priori knowledge of the structure of instantaneous causation, i.e., the time-series have to be ordered in a way that instantaneous effects are allowed from one process to another but not vice versa. The instantaneous effects were ordered based on information of the physiological system: respiration was measured before ARI and before SBP due to the pulse transit time and the time delay between electrical and mechanical activation.

## Appendix B: DEFINITIONS OF SPECTRA, COHERENCE, AND DIRECTED COHERENCE FOR MVAR MODELS

The spectral representation of the MVAR process is derived considering the Fourier transform of the MVAR process:

Y(f)=B(f)Y(f)+W(f)

The transfer function that links input and output is the inverse of the coefficients of B:

$$H\left(f\right)={\left[I-B\left(f\right)\right]}^{-1}$$

where I is the identity matrix. The spectral matrix is then defined as:

$$S\left(f\right)=H\left(f\right)\wedge H^{H}\left(f\right)$$

where the superscript H stands for the Hermitian transpose and ∧ is the covariance matrix. Its compact form is written as:

$$Sij\left(f\right)=h_{i}\left(f\right)\wedge h_{j}\left(f\right)$$

Under the assumption that the input white noises are uncorrelated, their covariance matrix ∧ reduced to the diagonal matrix $\wedge =diag\left(\sigma_{i}^{2}\right)$, where $\sigma_{i}^{2}$ is the variance of w_i_.

S can be factorized in:

$$S_{ij}\left(f\right)=\overset{3}{\underset{m\;=1}{\sum }}\sigma_{m}^{2}H_{im}\left(f\right)H_{jm}^{*}\left(f\right)$$

The coherence between y_i_ and y_j_ is defined as:

$${ϒ}_{ij}\left(f\right)=\frac{S_{ij}\left(f\right)}{\sqrt{S_{ii}\left(f\right)S_{jj}\left(f\right)}}$$

$${ϒ}_{ij}\left(f\right)=\frac{h_{i}\left(f\right)\sum h_{j}^{H}\left(f\right)}{\sqrt{h_{i}\left(f\right)\sum h_{i}^{H}\left(f\right)}\sqrt{h_{j}\left(f\right)\sum h_{j}^{H}\left(f\right)}}=\overset{3}{\underset{m\;=1}{\sum }}\frac{\sigma_{m}H_{im}\left(f\right)}{\sqrt{S_{ii}\left(f\right)}}\frac{\sigma_{m}H_{jm}^{*}\left(f\right)}{\sqrt{S_{jj}\left(f\right)}}=\overset{3}{\underset{m\;=1}{\sum }}\gamma_{im}\left(f\right)\gamma_{jm}^{*}\left(f\right)$$

The last term contains the so-called directed coherence. The directed coherence (γ) from y_i_ to y_j_ is defined as:

$$\gamma_{ij}\left(f\right)=\frac{\sigma_{j}H_{ij}\left(f\right)}{\sqrt{\sum_{m\;=1}^{3}\sigma_{m}^{2}{\left|H_{im}\left(f\right)\right|}^{2}}}$$

## Appendix C: METHODOLOGICAL CONSIDERATIONS FOR FREQUENCY DOMAIN MEASURES OF CAUSALITY

For this study, respiration was required to be voluntarily controlled, and the tidal change voluntarily adjusted to suit the wide range of rates, thus avoiding hyper- or hypoventilation. By controlling the respiratory frequency, we endeavored to maximize stationarity of respiratory modulation of APD. Estimation of the amplitude using the power spectra are, therefore, likely to have produced the average frequency content of the ARI and SBP signal over the entire recording time of 90 s. To provide a good representation of the frequency content, Thomson's multitaper spectra were used to estimate the amplitude of ARI and SBP oscillations. When compared with the Fourier Transform, this method reduces the amplitude estimation bias by obtaining multiple estimates from the same sample and averaging over all the tapered spectra (48). The cyclical variation of ARI and SBP was quantified as peak-to-peak amplitude, since this measure represents the absolute changes in APD.

Frequency domain measures of causality have been used previously to investigate causal interactions between spontaneous variability of the heart period (RR interval), SBP, and the respiratory flow (36, 39). To the best of our knowledge, our work is the first application in studying cardiorespiratory interactions between ventricular endocardial APD, SBP, and RESP. In this work, we used a MVAR model that describes both lagged and instantaneous effects as proposed by Faes and colleagues (12, 38, 39). The use of a strictly causal MVAR models to describe multiple time series with zero-lag correlations may lead to incorrect estimates of the lagged effects and thus to erroneous causality inferences.

MEC is the process by which mechanical forces on the myocardium can alter its electrical properties (3, 27, 29, 42, 47). In contrast with the baroreceptor reflex, MEC may act almost instantaneously (i.e., before arrival of the next heart beat). Consequently, to achieve a full description of the correlation structure of the observed signals, we used an extended MVAR model that combines both instantaneous and lagged effects. When neglecting instantaneous effects, we discovered cross-correlation between the residuals, indicating that causality measures would have been adversely affected when excluding instantaneous effects (12, 13). The analysis showed that the directed coherence from ARI to RESP and from SBP to RESP was (very) low, which corresponds to our expectations, since these pathways do not have any physiological meaning. With respect to nonparametric methods, MVAR analysis presents the advantage of infer directionality and causality through the parameterization of the mutual interactions between processes (37).
